# The (in)effectiveness of anticipatory vibrotactile cues in mitigating motion sickness

**DOI:** 10.1007/s00221-023-06596-8

**Published:** 2023-03-27

**Authors:** A. J. C. Reuten, J. B. J. Smeets, J. Rausch, M. H. Martens, E. A. Schmidt, J. E. Bos

**Affiliations:** 1 Department of Human Movement Sciences, Vrije Universiteit Amsterdam, Amsterdam, The Netherlands; 2 Human Performance, The Netherlands Organization for Applied Scientific Research (TNO), Soesterberg, The Netherlands; 3 Ford Research and Innovation Center, Aachen, Germany; 4 Traffic and Transport, The Netherlands Organization for Applied Scientific Research (TNO), The Hague, The Netherlands; 5 Department of Industrial Design, Eindhoven University of Technology, Eindhoven, The Netherlands

**Keywords:** Time-dependence, Car sickness, Neural mismatch theory, Internal model, Self-driving cars

## Abstract

The introduction of (fully) automated vehicles has generated a re-interest in motion sickness, given that passengers suffer much more from motion sickness compared to car drivers. A suggested solution is to improve the anticipation of passive self-motion via cues that alert passengers of changes in the upcoming motion trajectory. We already know that auditory or visual cues can mitigate motion sickness. In this study, we used anticipatory vibrotactile cues that do not interfere with the (audio)visual tasks passengers may want to perform. We wanted to investigate (1) whether anticipatory vibrotactile cues mitigate motion sickness, and (2) whether the timing of the cue is of influence. We therefore exposed participants to four sessions on a linear sled with displacements unpredictable in motion onset. In three sessions, an anticipatory cue was presented 0.33, 1, or 3 s prior to the onset of forward motion. Using a new pre-registered measure, we quantified the reduction in motion sickness across multiple sickness scores in these sessions relative to a control session. Under the chosen experimental conditions, our results did not show a significant mitigation of motion sickness by the anticipatory vibrotactile cues, irrespective of their timing. Participants yet indicated that the cues were helpful. Considering that motion sickness is influenced by the unpredictability of displacements, vibrotactile cues may mitigate sickness when motions have more (unpredictable) variability than those studied here.

**Supplementary Information:**

The online version contains supplementary material available at 10.1007/s00221-023-06596-8.

## Introduction

All individuals with functioning organs of balance are susceptible to motion sickness (Irwin 1881; James 1883). It is a syndrome of discomfort with symptoms such as dizziness, headaches, nausea and vomiting (Money 1970). The earliest reports date back hundreds of years, with narratives of sea-sickness, cart-sickness, and camel-sickness documented in ancient literature (Brandt et al. 2016; Huppert et al. 2017). Many have ever since attempted to explain its origin, and foremost, the ways to mitigate it (e.g., Lackner 2014; Golding 2016).

The neural mismatch theory identified the root cause of motion sickness as a mismatch between sensory signals on self-motion and estimations, predictions, or expectations thereof (Reason and Brand 1975; Reason 1978; Oman 1991). Improving these expectations would hence offer a way to mitigate motion sickness. The easiest solution then seems to provide someone control of self-motion, as was demonstrated by Rolnick and Lubow (1991). They reported that participants in control of their head motion reported less motion sickness compared to participants passively exposed to the same stimulus. This could explain why car drivers suffer less from sickness compared to car passengers (Schmidt et al. 2020). The introduction of (fully) automated vehicles thereby comes with an additional challenge. As their essence is to eliminate human interference with driving, their usage is inherently paired with an expected increase in motion sickness prevalence (reviewed by Iskander et al. 2019). The aim of our study is to investigate the effectiveness of a potential solution.

Helping individuals to anticipate certain vehicle motions has shown to be a promising solution to mitigate motion sickness. This anticipation can be provided via *anticipatory cues* which alert occupants of changes in the upcoming motion trajectory via vision (Feenstra et al. 2011; Hainich et al. 2021; Karjanto et al. 2018) or sound (Kuiper et al. 2020a; Diels and Bos 2021; Maculewicz et al. 2021). However, visual cues sometimes aggravate a neural mismatch, provoking rather than mitigating motion sickness (Stauffert et al. 2020; Karjanto et al. 2021). Furthermore, the opportunity to engage in non-driving related tasks already occupying the visual or auditory system (Kyriakidis et al. 2015) could result in occupants missing a cue (Lerner et al. 2015; Meng and Spence 2015) or feeling disturbed by it (Diels and Bos 2021). As an alternative, anticipatory cues could be presented via a third channel unaffected by these disadvantages: the tactile modality. Vibrotactile cues are less intrusive whilst they are still hard to ignore and attention capturing (Scott and Gray 2008; Prewett et al. 2012; Petermeijer et al. 2016). Tactile displays have been used to augment human–machine interaction, for example to improve communication and navigation in the military or to recover from spatial disorientation during flight (Bos et al. 2005b; Hancock et al. 2015). Vibrotactile cues have also been successfully implemented in driver assistance systems such as navigation, lane keeping, and collision avoidance (Petermeijer et al. 2015; Gaffary and Lécuyer 2018). In this current study, we will investigate whether anticipatory vibrotactile cues can successfully mitigate motion sickness when being passively exposed to motion sickening displacements.

As far as our knowledge concerns, three studies have investigated the use of anticipatory vibrotactile cues for lateral displacements. Yusof et al. (2020) found no significant effect on motion sickness, whilst Karjanto et al. (2021) and Li and Chen (2022) reported a significant reduction. However, for the two studies that reported significant beneficial effects, we think their results have limited validity. First, the intervention used in Karjanto et al. (2021) was very similar to the one used by Yusof et al. (2020), except that it not only consisted of vibrotactile cues, but also included movable plates that pushed the participant’s upper body into the direction of a turn. Actively tilting head position into the centripetal force has been demonstrated to reduce motion sickness (Golding et al. 2003; Wada et al. 2012; Wada and Yoshida 2016). Given that the vibrotactile cues used in the study of Yusof et al. (2020) were not effective, the reduction of motion sickness in the study of Karjanto et al. (2021) might be attributed to the moving plates. Second, Li and Chen (2022) asked participants to indicate the direction of anticipated car motion by steering the wheel into the direction of the perceived vibration. Some participants afterwards expressed to have felt in control of the vehicle’s motion. As control of self-motion is hypothesized to strongly reduce motion sickness (Rolnick and Lubow 1991), the finding of Li and Chen (2022) might not be due to the cue itself. Furthermore, in both studies the reported levels of motion sickness were rather low, which may make one wonder if these studies succeeded in provoking motion sickness at all. Overall, we think that the evidence on the effectiveness of purely anticipatory vibrotactile cues is yet inconclusive.

In this study, we will re-evaluate the effectiveness of vibrotactile cues only for mitigating motion sickness caused by longitudinal displacements. If we can confirm their effectiveness, a next question would be how much time in advance of motion onset they should be presented. Our research question is thus twofold: first, we question whether anticipatory vibrotactile cues successfully mitigate motion sickness, and second, which of our selected anticipatory intervals between the cue and motion onset is most effective. To that end, we exposed participants to four sessions of sickening motion that differed in the timing of vibrotactile stimulation. We hypothesized that the anticipatory vibrotactile cues would mitigate motion sickness, though we had no expectations which anticipatory interval would be most effective.

## Methods

To investigate whether the effectiveness of anticipatory vibrotactile cues is dependent on their timing, we examined self-reported motion sickness in four sessions. These sessions only differed in the anticipatory time interval between a vibrotactile cue and motion onset of a linear sled. In three sessions, the cue was predictive and alerted participants of the onset of a displacement. We compared motion sickness in these anticipatory sessions to that in a control session, in which the cue was only presented until after the onset of motion. We preregistered our study on the Open Science Framework (https://doi.org/10.17605/OSF.IO/SYVU9).

### Participants

Our aim was to have a fully counterbalanced within-subjects design, which required 24 participants to complete all four sessions. Accounting for dropouts, we set our recruitment criterion at 30 participants. To be included in our study, participants had to be 18 years or older, experienced car sickness in the last five years, and free of self-known vestibular disorders. Participants additionally had to be in good health according to self-report, for example not suffering from cardiovascular or neurological disorders. After being recruited, 10 participants could not be included in the results because of no-show (*n* = 7), a severe motion sickness response resulting in the decision to cancel participation (*n* = 2), or mechanical failure of the device (*n* = 1). This left 20 participants to complete all sessions, which sample size should provide sufficient statistical power when comparing to similar experiments reporting significant effects (e.g., Feenstra et al. 2011; Kuiper et al. 2020a). Participants were aged between 18 and 61 years (*M* = 26 years, 17 females), the majority being students from the Vrije Universiteit Amsterdam. We have obtained ethical approval from the institutional review board of TNO, which is the organization where the experiment was performed.

### Motion stimuli

**Fig. 1:**
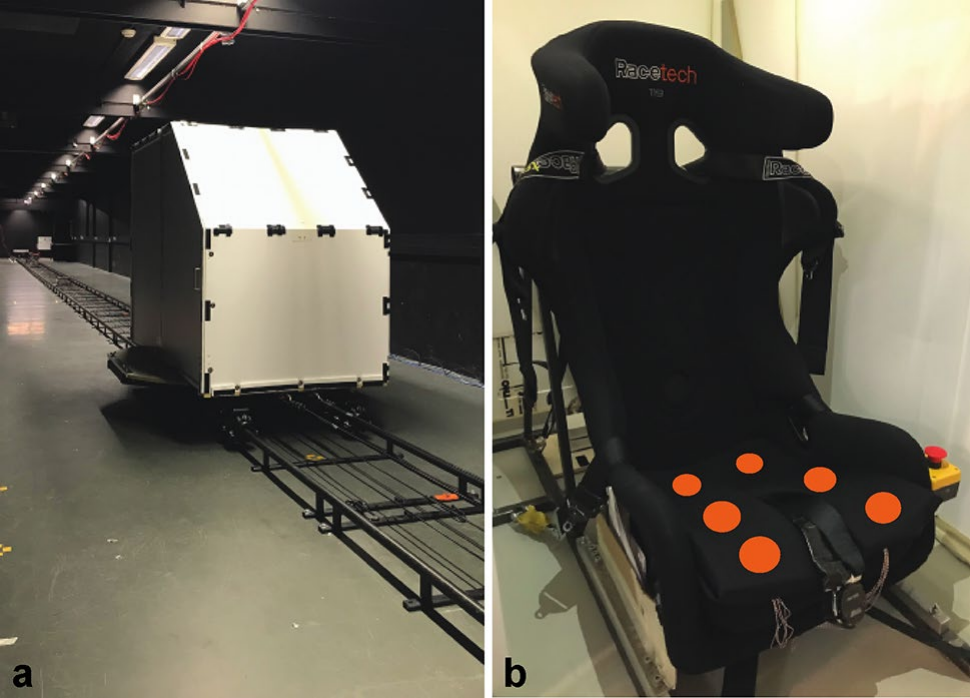
**a** The linear sled that was used in this study. The illuminated cabin offered an enclosed space that removed external visual and airflow cues. **b** Interior view of the cabin where the participants were seated. The stationary visual frame of reference provided by the cabin resembles the context of a car ride without looking outside. A printed version of the used motion sickness scale was taped onto the wall in front of the participants. Participants could also see a webcam which was used for observation. The rally seat offered a head rest and a five-point seat belt for safety. The orange dots indicate the position of the six vibrotactile actuators

In each session, we exposed participants to a series of 65 sickening fore-aft displacements on a linear sled (Fig. 1a). This linear sled is ideally suited to consistently produce linear accelerations which succeed one another rapidly. We used the displacements by Kuiper et al. (2020a) as a starting point for defining our motion stimulus. Because we here wanted to isolate the effect of the anticipatory interval, we used displacements predictable in direction that all followed an identical asymmetrical acceleration profile (see Supplementary Fig. S1). Each displacement consisted of a fast forward motion (peak acceleration 3.5 m/s^2^) followed by a deceleration leading to a slow (theoretically unprovocative) backward motion at constant velocity. This asymmetry ensured the most provocative part of the displacement was closest to the anticipatory cue. The fore and aft motion took about 9 s in total. The amplitude of each displacement was 7.2 m, with the cabin repeatedly returning to its starting position. The start of consecutive displacements was randomly varied between 12 and 20 s according to a uniform distribution, making it impossible for participants to reliably predict the onset of the displacement without an anticipatory cue. This type of motion somewhat resembles driving in a traffic jam, with short forward accelerations at inconsistent intervals. As inertial motion with constant velocity cannot be perceived, the stationary intervals could also represent intervals of any constant velocity during a real car ride, with the displacements representing periods of acceleration and deceleration. We generated four variations of the series of displacements and stationary intervals, and exposed all participants to each variation once, with all variations equally distributed across sessions. The exposure duration was 15 min per session, which is comparable to the duration used in other cueing studies (e.g., Kuiper et al. 2020a; Feenstra et al. 2011; Hainich et al. 2021).

### Vibrotactile cues

**Fig. 2:**
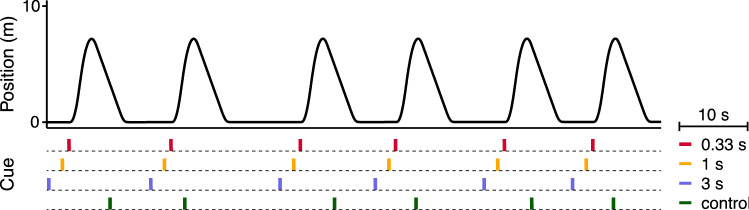
Schematic overview of the timing of vibrotactile stimulation relative to the onset of motion in the four sessions

We presented the vibrotactile cues by means of six small (approximately 5 × 20 mm) eccentric rotatory mass vibration motors embedded horizontally in a 2-cm foam cushion placed on top of the seat pan (Fig. 1b). The cue consisted of simultaneously activating the six actuators at 125 Hz for a duration of 150 ms. In three anticipatory sessions, the onset of the cue was always *prior* to the onset of forward motion: either at 0.33, 1, or 3 s. We selected these three equidistant anticipatory intervals, because previous cueing studies used intervals within this range (de Winkel et al. 2021; Diels and Bos 2021; Hainich et al. 2021; Karjanto et al. 2018, 2021; Kuiper et al. 2020a; Li and Chen 2022; Maculewicz et al. 2021; Yusof et al. 2020). To account for any effect of the cue itself (rather than its predictive information), we included a control session in which the onset of a non-informative cue was 2–6 s *after* the onset of forward motion. We chose this variable interval to minimize any predictability associated with this cue, equal to the interval selected by Kuiper et al. (2020a). The presentation of vibrotactile cues in relation to the displacements is visualized in Fig. 2. The order of sessions was counterbalanced and then randomly assigned to participants.

### Measures

We quantified the progression of motion sickness by asking the participants for a Motion Illness Symptoms Classification score (MISC; see Table 1; developed by Bos et al. 2005a; elaborated on and renamed by Reuten et al. 2021) at 1 min intervals in each of the four sessions. We also asked participants to fill out the Motion Sickness Susceptibility Questionnaire (MSSQ-Short; Golding 2006) and a self-developed user experience questionnaire. After each session, we asked participants if and when they felt the cues (multiple-choice, Fig. 6a); how often they felt the cues (multiple-choice, Fig. 6b); and how they evaluated the cues along a range of user dimensions (Likert-scale, Fig. 6c). After the fourth session, we asked participants if they noticed that the cues in each session were presented at fixed times relative to the start of the displacements (multiple-choice, see text), which cue they preferred in announcing the onset of motion (multiple-choice, Fig. 6d), if they would want to use that cue in their (autonomous) car (multiple-choice, Fig. 6e), how much money they would be willing to spend extra on a car preventing motion sickness (open-ended, see text), and if they had suggestions to adjust the cue (open-ended, see text).

**Table 1:**
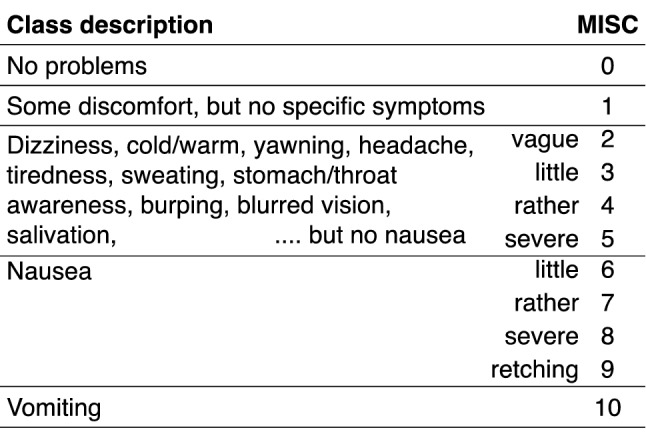
The Motion Illness Symptoms Classification (MISC) used to assess motion sickness symptomatology

### Procedure

Participants performed the four sessions divided across two days. On the first day, participants received instructions on the experimental procedure and signed an informed consent form. They subsequently filled out the MSSQ-Short (Golding 2006), from which we observed that the susceptibility towards motion sickness of our 20 participants corresponds to the 76th percentile. We instructed participants that our study was on the effectiveness of vibrotactile cues in mitigating motion sickness, and that a vibrotactile cue would be presented prior to the sled’s forward motion in some sessions, and during the motion in other sessions. Participants subsequently performed a familiarization trial of three displacements (< 1 min; see Motion Stimuli) without vibrotactile stimulation, followed by a 10 min break. They then performed two out of the four sessions, with a 1 h break in between to recover from any motion sickness. To control for carry-over effects, participants performed the remaining two sessions 7 days later. This period was extended for five participants (mainly due to the COVID-19 virus): 3 participants performed the sessions 14 days later, 1 participant 22 days later, and 1 participant 42 days later.

Participants could only start a session when they rated a MISC score of 0 or 1 at the start of the session (i.e., *t* = 0). Two participants rated a higher pre-test MISC score, wherefore we aborted the experiment for one participant and waited until the symptoms disappeared for another participant. During the sessions, we could observe the participant via a video connection, and remained in contact via a two-way audio connection. We asked participants to perform an auditory 1-back task to control their focus of attention, in which they needed to count the number of duplicate vowels heard. We also instructed participants to keep their eyes open and head upright. If they rated MISC ≥ 6, we aborted the session. After each session and at the end of the experiment, we asked participants to fill out a user experience questionnaire. They received study credits or a monetary reward for their participation in the experiment.

### Data analysis

To determine the effect of the anticipatory vibrotactile cues, we developed a way to express their effectiveness into a single value that captured the difference in the development of motion sickness between each of the anticipatory sessions relative to the control session. This value is meaningful when the cue provides a constant effect during a session. We tested our approach with data obtained in a similar experiment by Kuiper et al. (2020a), who presented an auditory cue before (anticipatory session) or after (control session) motion onset of a linear sled. In this section, we illustrate our analysis method using their data.

Assuming a positive effect of the anticipatory ($A$) session relative to the control ($C$) session, we first calculate the reduction ${R}_{ti}$ of MISC scores per time point ($t$) and individual participant ($i$) by

$${R}_{ti}=\frac{\left({C}_{ti}-{A}_{ti}\right)}{\left({C}_{ti}+{A}_{ti}\right)}$$

We use the measure $R$ instead of a percentage change (i.e., $S=(1- A/C)\times 100$), because for ${R}_{ti}$ exchanging $C$ and $A$ only results in a change of sign. This makes it suitable for averaging: if $C$ and $A$ are drawn from a random distribution, the average of $R$ will be zero, whereas the average of $S$ will become negative. To provide the reader guidance on the interpretation of our measure*,* we provide a conversion of $R$ to a percentual change in MISC scores in Supplementary Fig. S2.

**Fig. 3:**
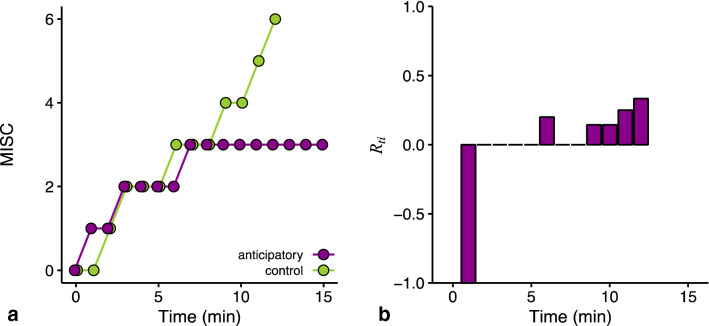
The initial steps of our method illustrated using data from participant 12 of Kuiper et al. (2020a). **a** The development of MISC scores. **b** The reduction ${R}_{ti}$ that results from the MISC scores in a). ${R}_{ti}$ has a low resolution for the first time points, with values either being −1 or 0

When ${C}_{ti}={A}_{ti}=0$, ${R}_{ti}$ becomes undefined. This is not problematic for our analysis as we will weigh the data as explained below; this undefined ${R}_{ti}$ value will receive a weight of zero. The range of possible $R$ values is symmetrical around zero (no reduction), ranging from −1 (maximum worsening, ${A}_{ti} \ne 0, {C}_{ti}=0$) to +1 (maximum mitigation, ${A}_{ti}=0, {C}_{ti}\ne 0$), see also Supplementary Fig. S3. One of the advantages of our measure $R$ is that we can determine the effectiveness of the cue for each of the 15 time points within a session. Because participants only rate MISC 0 or 1 early on in a session, the resolution of ${R}_{ti}$ is low for the first time points: ${R}_{ti}$ will either be 0, 1, or −1. This consequence is visualized in Fig. 3, where we present the MISC scores (a) and resulting ${R}_{ti}$ values (b) for one participant. Note that we do not calculate ${R}_{ti}$ at *t* = 0 (pre-test measurement), and cannot determine ${R}_{ti}$ for those time points with a missing MISC score as the result of the exerted stop-criterion.

To take the resolution of ${R}_{ti}$ into account when determining the average reduction of the cue, we weight (${w}_{ti}$) each of the 15 obtained ${R}_{ti}$ values by the sum of the two underlying MISC scores

$${w}_{ti}={C}_{ti}+{A}_{ti}$$

We can then calculate the average reduction per participant $i$ and for each time point $t$ by

$${\overline{R} }_{i}=\frac{\sum_{t}{w}_{ti}{R}_{ti}}{\sum_{t}{w}_{ti}}=\frac{\sum_{t}({C}_{ti-}{A}_{ti})}{\sum_{t}{w}_{ti}}$$

and

$${\overline{R} }_{t}=\frac{\sum_{i}{w}_{ti}{R}_{ti}}{\sum_{i}{w}_{ti}}=\frac{\sum_{i}{(C}_{ti-}{A}_{ti})}{\sum_{i}{w}_{ti}}$$

The first equation indicates that ${\overline{R} }_{i}$ is proportional to the difference between the two sessions (i.e., the area between the two curves in Fig. 3a).

**Fig. 4:**
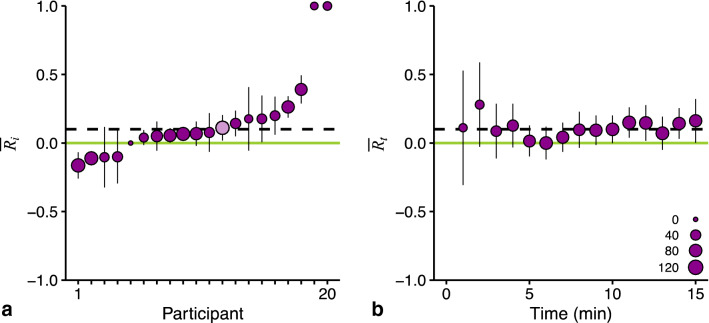
Our method to determine the reduction ($R$) of motion sickness illustrated with data from Kuiper et al. (2020a). **a** The average for individual participants ($i$), who are ordered based on the size of $\overline{{R }_{i}}$. Participant 12 (data point in light purple) was the example participant whose data we presented in Fig. 3. **b** The average for each time point ($t$). For both panels, the averages are weighted based on the sum of MISC scores underlying the data. The size of the points reflects the sum of these weights (see legend in panel b). The line in light green corresponds to no reduction (i.e., $R=0$). The dashed line represents the overall reduction $\overline{R }$ in this experiment. The error bars are 95% confidence intervals calculated with bootstrapping of ${R}_{ti}$ and corresponding weights

Fifteen of the 20 participants in Kuiper et al. (2020a) showed a reduction by the cue (${\overline{R} }_{i}>0$, Fig. 4a). Across the whole experiment, the reduction is fairly constant (none of the data-points in Fig. 4b deviates by more than its confidence interval), which supports our approach to use the MISC scores during the whole session to capture the reduction in motion sickness by a single number. We hence express the effectiveness of the cue across all time points and participants, again weighted by considering the resolution of ${R}_{ti}$ in

$$\overline{R }=\frac{\sum_{t}\sum_{i}{w}_{ti}{R}_{ti}}{\sum_{t}\sum_{i}{w}_{ti}}=\frac{\sum_{i}{w}_{i}{\overline{R} }_{i}}{\sum_{i}{w}_{i}}, \quad \mathrm{with }\,{w}_{i}=\sum_{t}{w}_{ti}$$

The resulting overall weighted average reduction is $\overline{R }$ = 0.10 (one-sided 95% confidence interval 0.02, ∞). The conclusion resulting from our new method of analysis corresponds with the original conclusion of Kuiper et al. (2020a): a significant reduction in motion sickness using anticipatory auditory cues.

### Statistical analysis

Our first question of interest is whether our anticipatory vibrotactile cues mitigate motion sickness. We therefore performed a weighted one-sided *t* test (with *α* = 0.05) to examine whether the grand mean of $\overline{R }$ across the three anticipatory sessions is larger than zero, with the grand mean of ${\overline{R} }_{i}$ of each participant weighted by the sum of their three ${w}_{i}$ scores. Our second question of interest is which of our selected time intervals between the anticipatory vibrotactile cue and motion onset mitigates motion sickness best. We therefore performed a weighted repeated measures ANOVA (*α* = 0.05) on the ${\overline{R} }_{i}$ values (each weighted by their respective ${w}_{i}$) of the three anticipatory sessions (0.33, 1, and 3 s).

All other analyses are not part of our pre-registration and should therefore be considered exploratory. To express the confidence of our estimates of $R$, we report two-sided 95% confidence intervals by default. When interested in whether $R$ was larger than zero, we instead report one-sided 95% confidence intervals using the format (lower bound, ∞).

## Results

**Fig. 5:**
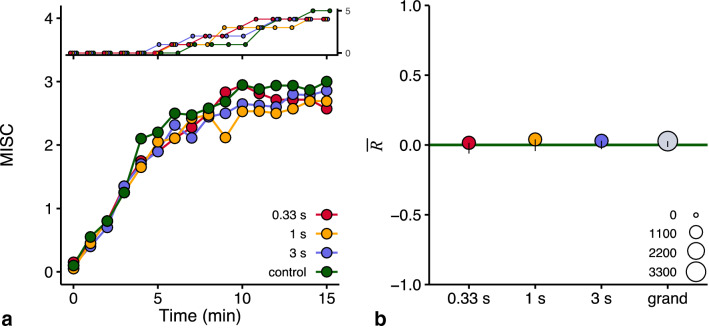
**a** The development of raw MISC scores averaged across participants for each of the four sessions. To enable a better comparison to Fig. 5b, we excluded data on those time points where participants reached the stop-criterion of MISC ≥ 6 in the control session. The inset figure displays the number of participants reaching the stop-criterion per time point. **b** The overall reduction ($\overline{R }$) in motion sickness generated by each anticipatory cue and their combined grand mean in gray. The line in dark green corresponds to no reduction. The size of the data points reflects the sum of MISC scores underlying the data (the overall weight, see legend). The error bars are one-sided 95% confidence intervals (coherent with our one-sided analysis) calculated with bootstrapping of ${\overline{R} }_{i}$ and corresponding weights

Our first question of interest is whether our anticipatory vibrotactile cues mitigated motion sickness. The pattern of MISC scores in Fig. 5a suggests a slight advantage for the anticipatory cues (see Supplementary Figs. S4–S5 for more details). We used our pre-registered analysis to quantify the effectiveness of each anticipatory cue by calculating $R$ (see Methods). As ${\overline{R} }_{t}$ did not vary systematically across the 15 time points within the sessions (see Supplementary Fig. S6), we only provide the overall reductions $\overline{R }$ per session (Fig. 5b). In line with visual inspection of this figure, a weighted one-sided *t* test confirmed that the grand mean of $\overline{R }$ across the three anticipatory sessions was not larger than zero (grand $\overline{R }$ = 0.03, *t* = 0.79, *p* = 0.22, 95% confidence interval -0.01, ∞). Our second question of interest is which of our selected anticipatory intervals between the cue and motion onset is most effective. A weighted repeated measures ANOVA indicated there was no difference between the ${\overline{R} }_{i}$ values of the three anticipatory sessions (*F*(2,51*)* = 0.13, *p* = 0.88). Under the chosen experimental conditions, our results did not show a significant mitigation of motion sickness by the anticipatory vibrotactile cues, irrespective of their timing. The $R$ values of the individual sessions can be found in Supplementary Fig. S6. To explore the existence of an order effect, we compared the MISC scores in the second, third, and fourth session to those rated in the first session. There is a tendency for the MISC scores to decrease with the greater number of sessions performed, though all confidence intervals included zero; suggesting no effect of session order (Supplementary Fig. S7).

**Fig. 6:**
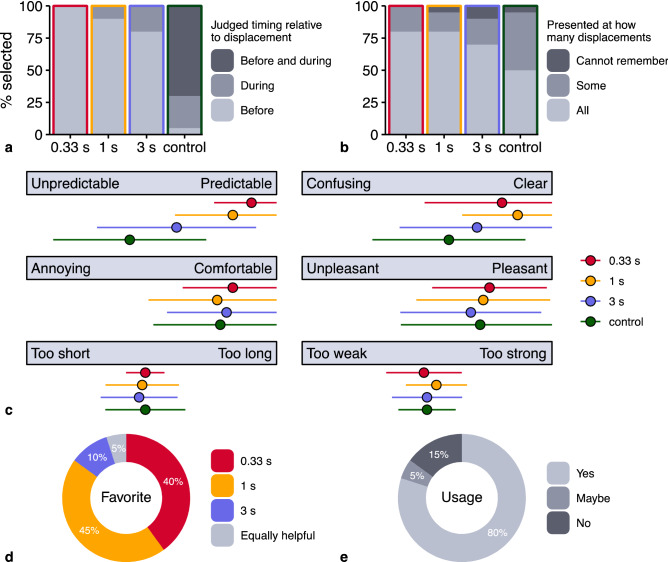
Results of the user experience questionnaire. Participants indicated **a** when they thought the cues were presented (the answer option “Not at all” not being selected), **b** how often they felt the cues, **c** how they evaluated the cues along a range of user dimensions (error bars indicate standard deviations), **d** which type of cue they preferred in announcing upcoming displacements (the answer options “None” and “Cannot remember” not being selected), and **e** if they would want to use the cue of their preference in their (autonomous) car

Using the results of the user experience questionnaire, we first wanted to confirm if participants noticed the cues and could correctly identify when they were presented. All participants noticed them, and the majority indeed indicated that the cues were presented prior to the onset of the displacement in the anticipatory sessions and during the displacement in the control session (Fig. 6a). Noticeable is a decreasing accuracy with longer anticipatory intervals. We also asked participants if they noticed that the cues were presented at a fixed moment relative to the onset of the displacements. All except for one participant did, with 50% of participants being aware of this in all sessions and 45% in some of the sessions. When questioning how often participants felt the vibrations, about 75% indicated to have felt them for every displacement in the anticipatory sessions (Fig. 6b). This percentage was considerably lower in the control session, possibly indicating that participants paid less attention to this cue as it did not have any anticipatory value.

The cues in the 0.33 s and 1 s anticipatory sessions were rated the most helpful to predict the onset of upcoming displacements (Fig. 6c). As was intended, the cue in the control session was rated the least helpful. All cues were furthermore rated positively in terms of pleasantness and comfort. Even though their duration and intensity were judged as appropriate, the few suggestions to improve the cue were mainly targeted at modification of these two aspects.

We also asked which anticipatory interval participants preferred in announcing the upcoming displacements (Fig. 6d). The 1 s interval was favored by most participants, followed by the 0.33 s interval. Several participants explicitly reported that the 3 s interval was too long, which complicated the exact estimation of motion onset. In congruence with those reasons, it was the least preferred cue with only 10% of all votes.

Four-fifths of the participants indicated they would want to use the cue they preferred in their (autonomous) car if it proved effective in mitigating motion sickness (Fig. 6e). There was a lot of variation in the amount of money participants were willing to spend extra on a car preventing motion sickness (*SD* = €744), with an average amount of €691. The three participants who indicated they would not want to use a cue reported they only suffered mild motion sickness and did not deem its use necessary.

## Discussion

We here investigated whether anticipatory vibrotactile cues are effective in mitigating motion sickness. We were also interested whether the timing of the cue influences its effectiveness. To that end, we exposed participants to four sessions of fore-aft motion on a linear sled. In three sessions, an anticipatory cue was presented prior to the onset of forward motion, either at 0.33, 1, or 3 s. We compared the scores on a motion sickness scale given within these sessions to the scores given in a control session with a non-anticipatory cue presented 2 to 6 s after motion onset. In contrast to our expectations, we found no evidence that the anticipatory cues were significantly mitigating motion sickness, irrespective of their timing (Fig. 5). This conclusion following our newly defined method $R$ aligns with that of a more traditional analysis approach using a repeated measures ANOVA on the raw MISC scores, which we reported at a conference (Reuten et al. 2022).

For the anticipatory cues to work, participants should associate them with the upcoming displacement. A limitation of our study is that this might not have been easy in the session with a 3 s anticipatory interval, as the shortest interval between consecutive displacements was 4 s. This may explain why about a quarter of the participants indicated that the cue was presented both before and during (instead of only before) the displacements of this session (Fig. 6a). If we re-analyse the reduction of motion sickness including only those participants who correctly identified the timing of the cues, the confidence interval of the cue with the 3 s anticipatory interval does not include zero, which suggests that this cue mitigated motion sickness (see Supplementary Fig. S8a). However, given that this analysis was not pre-registered and only included twelve participants, this finding should be interpreted with caution. Moreover, the fact that the remaining participants rated the 3 s cue less helpful compared to the cues with shorter anticipatory intervals (see the user experience ratings in Supplementary Fig. S8b), contradicts the argument that linking the cue to the previous displacement is causing the lack of a significant reduction of motion sickness.

Another potential limitation of our study is that the linear sled sporadically deviated from the programmed motion stimulus, resulting in some displacements getting a bit jerky. This means that some part of the motion was not announced by the cues, which may explain why our results did not show a significant mitigation of motion sickness. At the same time, it can be reasoned that in a real-world scenario not all motions can correctly be predicted and accompanied by an appropriate anticipatory cue, so an ideal cue should be effective despite the presence of some unpredictable motion.

Kuiper et al. (2020a) performed a comparable study on the effectiveness of anticipatory auditory cues. They used the same linear sled as we used to subject 20 participants to a motion stimulus similar in provocativeness to ours (see Supplementary Fig. S9). The participants’ motion sickness susceptibility scores on the MSSQ were also comparable (76th versus 70th percentile). As we reported in our Methods section, our analysis method yields a significant advantage of the anticipatory auditory cue in that experiment, whereas the vibrotactile cue in this experiment did not. This may suggest superiority of the auditory modality over the tactile modality. However, two arguments challenge that suggestion. First, a weighted independent samples *t* test indicates there is no difference in the grand $\overline{R }$ = 0.03 of our study and $\overline{R }$ = 0.10 in Kuiper et al. (2020a), with *t* = 1.15 and *p* = 0.26. Though only the reduction in Kuiper et al. (2020a) was significantly larger than zero, this does not by definition imply that their intervention was more effective than ours. Such a conclusion requires a direct comparison, see the second common mistake in Makin and Xivry (2019). Second, the experiments differed in the variability of the displacements: we only varied the onset of the displacements, whereas Kuiper et al. (2020a) additionally varied their direction (forward or backward). Because unpredictability about motion onset and direction individually contribute to the motion sickness response (Kuiper et al. 2020b), the additional unpredictability of motion direction may explain why the cue in Kuiper et al. (2020a) was more effective compared to our study. These arguments necessitate a direct comparison between the effectiveness of auditory and vibrotactile cues. We will therefore re-evaluate the effectiveness of directional vibrotactile cues with displacements unpredictable in both onset and direction, together with a comparison of auditory cues in a follow-up study (pre-registered at 10.17605/OSF.IO/8FZU7).

Though our results did not provide evidence that anticipatory vibrotactile cues are effective in mitigating motion sickness, we think several reasons make it worthwhile to investigate how their effectiveness can be improved. First of all, despite the fact that our cues did not significantly reduce motion sickness, a comparison to the auditory cues of Kuiper et al. (2020a) indicated the vibrotactile cues were not performing significantly worse. Second, most of our participants indicated that the vibrotactile cues with short anticipatory intervals (i.e., 0.33 and 1 s) were helpful in announcing the onset of upcoming displacements, and also expressed the willingness to have them in their (autonomous) car. Lastly, the tactile modality seems specifically suited for usage in automated vehicles. For example, vibrotactile cues will not interfere with the non-driving related tasks passengers may want to perform. We will first re-evaluate if vibrotactile cues mitigate motion sickness when motions are harder to anticipate, in particular when considering changes in vehicle velocity in multiple directions as representative for real on-road driving, instead of only one as studied here. Other work could focus on including a training to familiarize with the cues or the additive effect of combining multiple mitigation approaches as studied by Karjanto et al. (2021). Alternatives are investigating the positioning of the actuators or the advantage of self-adjustable intensity settings to match individual preferences (Duthoit et al. 2018). Longer anticipatory time intervals might be studied as well, though previous cueing studies (e.g., Hainich et al. 2021; Karjanto et al. 2018; Kuiper et al. 2020a; Maculewicz et al. 2021) reported significant effects when using time intervals comparable to those studied here. Despite not finding a significant reduction in motion sickness, we still conclude it is worthwhile to elaborate further on the effectiveness of anticipatory vibrotactile cues in future research.

## Supplementary Information

Below is the link to the electronic supplementary material.

Supplementary file1 (PDF 316 KB)

## Data Availability

All data and code can be publicly accessed on the Open Science Framework (https://osf.io/bsznv/).
