# Supplementary material for: The (in)effectiveness of anticipatory vibrotactile cues in mitigating motion sickness
Source: Exp Brain Res. 2023 Mar 27;241(5):1251–61. doi: 10.1007/s00221-023-06596-8 (PMC10042112; doi:10.1007/s00221-023-06596-8)
Supplement: Supplementary file 1 — Supplementary file1 (PDF 316 KB) [file 221_2023_6596_MOESM1_ESM.pdf]

The (in)effectiveness of anticipatory vibrotactile cues in mitigating motion sickness

A. J. C. Reuten[1,2]*, J. B. J. Smeets[1], J. Rausch[3], M. H. Martens[4,5], E. A. Schmidt[3] & J. E. Bos[2,1]
[1] Department of Human Movement Sciences, Vrije Universiteit Amsterdam, Amsterdam, The Netherlands
[2] Human Performance, The Netherlands Organization for Applied Scientific Research (TNO), Soesterberg, The Netherlands
[3] Ford Research and Innovation Center, Aachen, Germany
[4] Traffic and Transport, The Netherlands Organization for Applied Scientific Research (TNO), The Hague, The Netherlands
[5] Department of Industrial Design, Eindhoven University of Technology, Eindhoven, The Netherlands

Supplementary Information

Motion stimulus

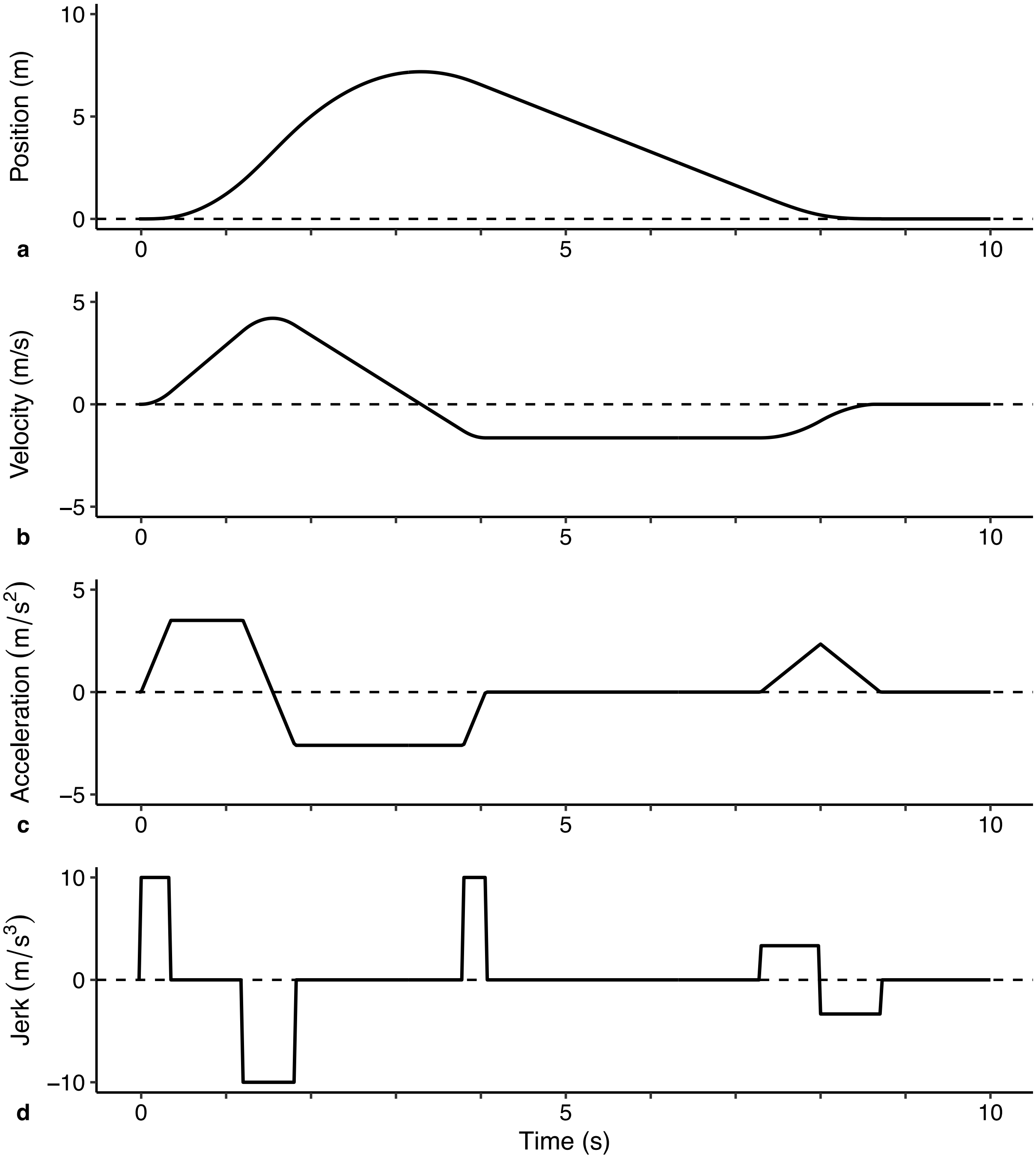

Fig. S1. Motion characteristics of one displacement

Interpretation of the Reduction (R) measure

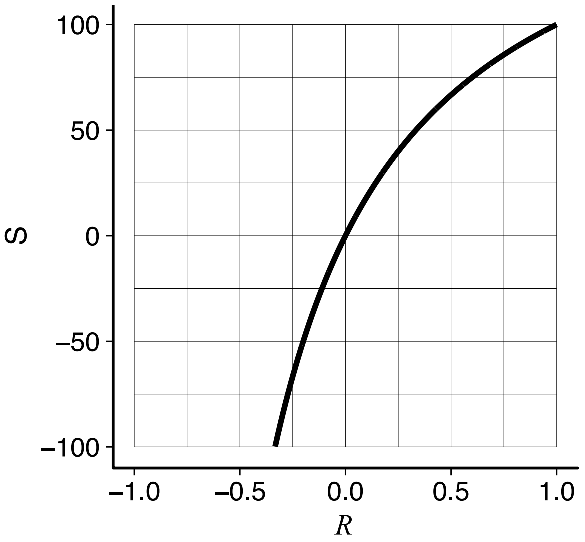

Fig. S2. Guidance to the interpretation of our measure R expressed in terms of the percentual change in MISC scores from the anticipatory to control session ($S = (1 - A/C) \times 100$). Note that because S is an asymmetrical measure, R values lower than -0.4 correspond to extremely large negative values of S

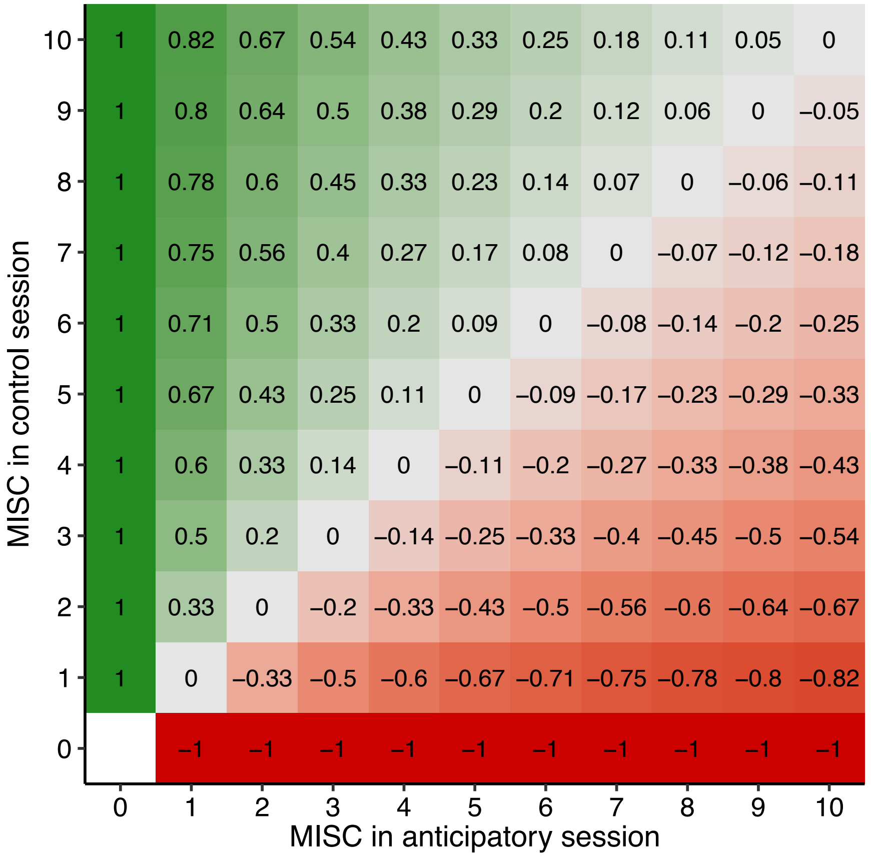

Fig. S3. Illustration of the distribution of possible R_{ti} values (see 'Methods' of the main text). When $C_{ti} = A_{ti} = 0$, R_{ti} becomes undefined. This does not interfere with our analysis as it will receive a weighting of 0

Temporal response traces for each participant

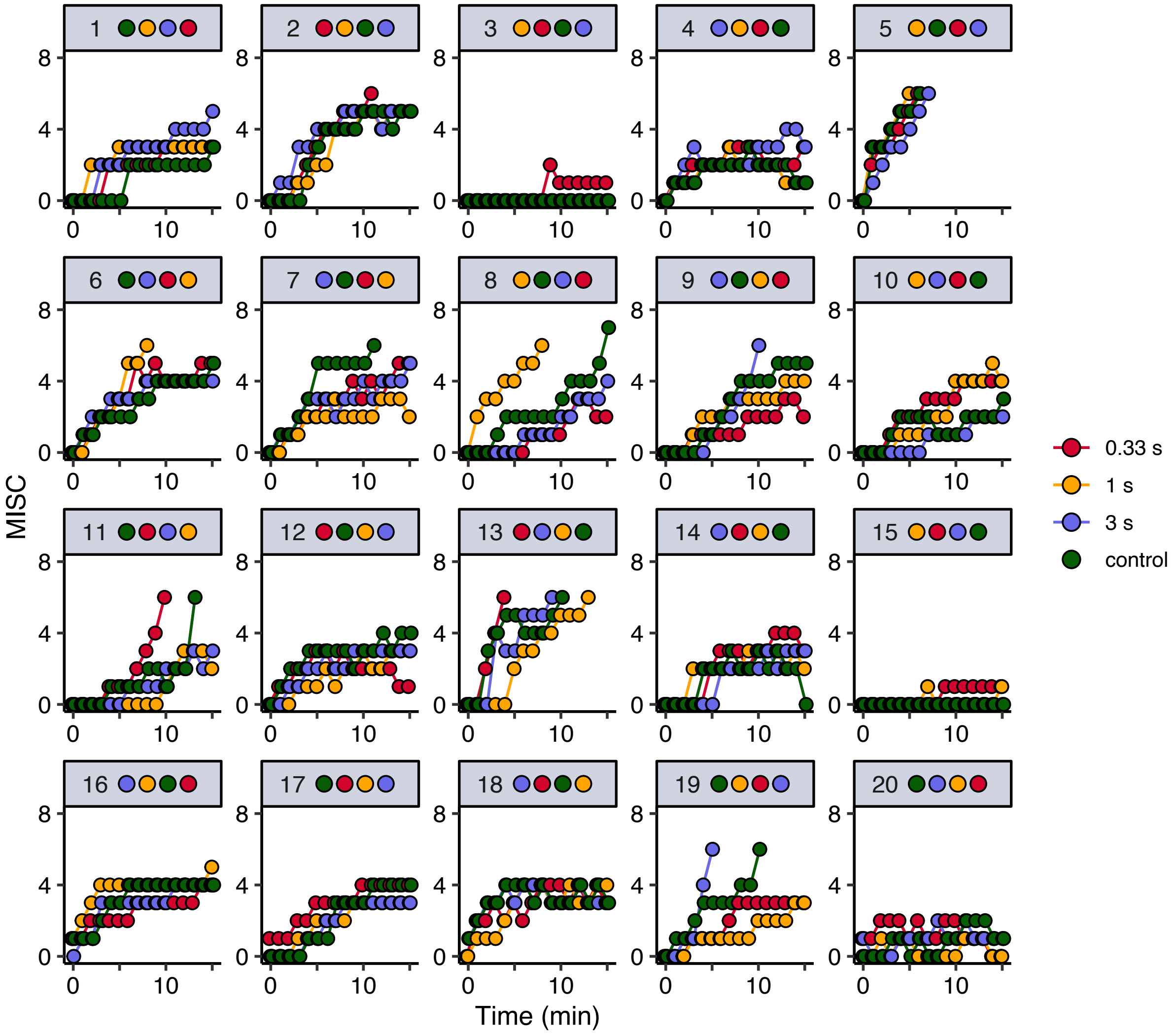

Fig. S4. The development of raw MISC scores as a function of the time in a session. The red, yellow and blue sessions are the three anticipatory sessions. The control session is presented in green. Each panel reflects the order of four sessions of a single participant

The development of motion sickness with replacement of missing data

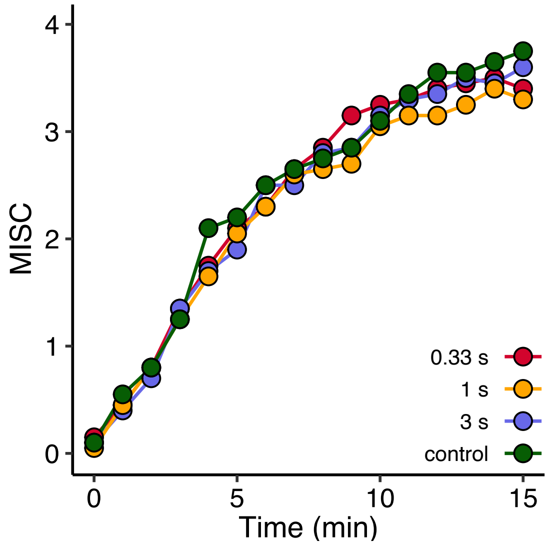

Fig. S5. The development of MISC scores averaged across participants for each of the four sessions. In comparison to our main analysis, we here replaced missing data as the result of the exerted stop-criterion at MISC 6 with the last rated MISC score. In agreement with Fig. 5a of the main text, the pattern of results again suggest a slight advantage for the anticipatory cues

Reduction (R) values per anticipatory session

Here we present the reduction (R) values per participant (i) and time point (t) for each of the anticipatory sessions. The R values reflect the effectiveness of the anticipatory cues. They express the amount of reduction in MISC scores from an anticipatory session (0.33 s, 1 s, 3 s) relative to the control session whilst accounting for the resolution of R. That is, each R value is weighted by the sum of MISC scores underlying the data. Positive R values indicate a reduction in motion sickness. The $\bar{R}_i$ values of participants 3 and 15 are small, because they reported no or only minimal symptoms of motion sickness (MISC $\leq$ 2). Their reduction values do accordingly not or only minimally contribute to the calculation of $\bar{R}$, which expresses the overall reduction per anticipatory session.

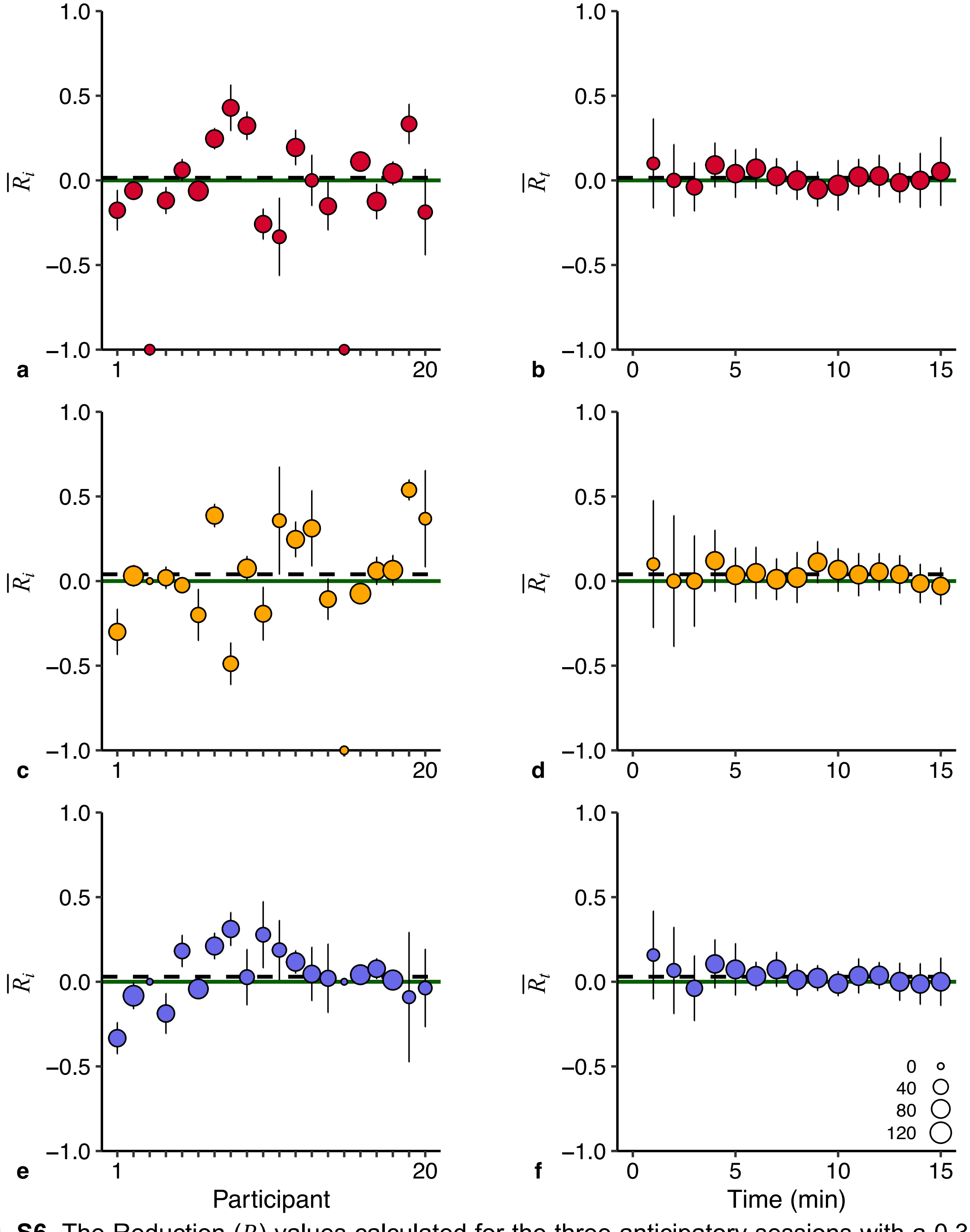

Fig. S6. The Reduction (R) values calculated for the three anticipatory sessions with a 0.33 s (in red), 1 s (in orange), and 3 s (in blue) time interval. **a**, **c**, **e)** The average for individual participants (i). **b, d, f)** The average for each time point (t). For all panels, the averages are weighted based on the sum of MISC scores underlying the data. The size of the data points reflects the sum of these weights (see legend in panel f). The line in dark green corresponds to no reduction (i.e., $R = 0$). The dashed lines represent the overall reduction $\bar{R}$ per anticipatory session. The error bars are 95% confidence intervals calculated with bootstrapping of R_{ti} and corresponding weights

Investigation of order effect

To explore the existence of an order effect, we compared the MISC scores in the second, third, and fourth session to those rated in the first session. There is a tendency for the MISC scores to decrease with the greater number of sessions performed. However, all confidence intervals include zero, suggesting the MISC scores did not deviate from those in the first session.

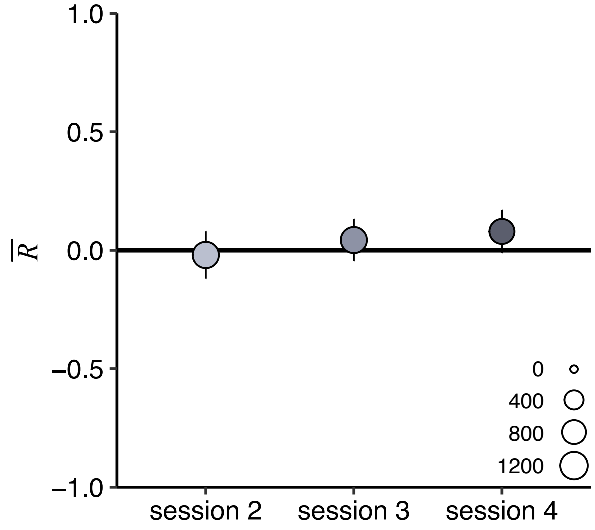

Fig. S7. The overall reduction ($\bar{R}$) in motion sickness calculated from the MISC scores provided in the second, third, and fourth session respective to the first session (black line). The black line hence corresponds to no difference in MISC scores. The size of the data points reflects the sum of MISC scores underlying the data (the overall weight, see legend). The error bars are 95% confidence intervals calculated with bootstrapping of $\bar{R}_i$ and corresponding weights

Re-analysis of Reduction (R) values based on subsample

We re-calculated the reduction (R) values based on a subsample including only those participants who correctly identified when the cues were presented (i.e., before motion onset in the anticipatory sessions and after motion onset in the control session, see Fig. 6a of the main text). Given that the lower bound of the 95% confidence interval of the cue with the 3 s anticipatory interval (0.02, ∞) does not overlap the dark green line (indicating no reduction), the results in Fig. S8a suggest this cue mitigated motion sickness. However, a re-analysis of the user experience ratings in Fig. S8b does not support that conclusion.

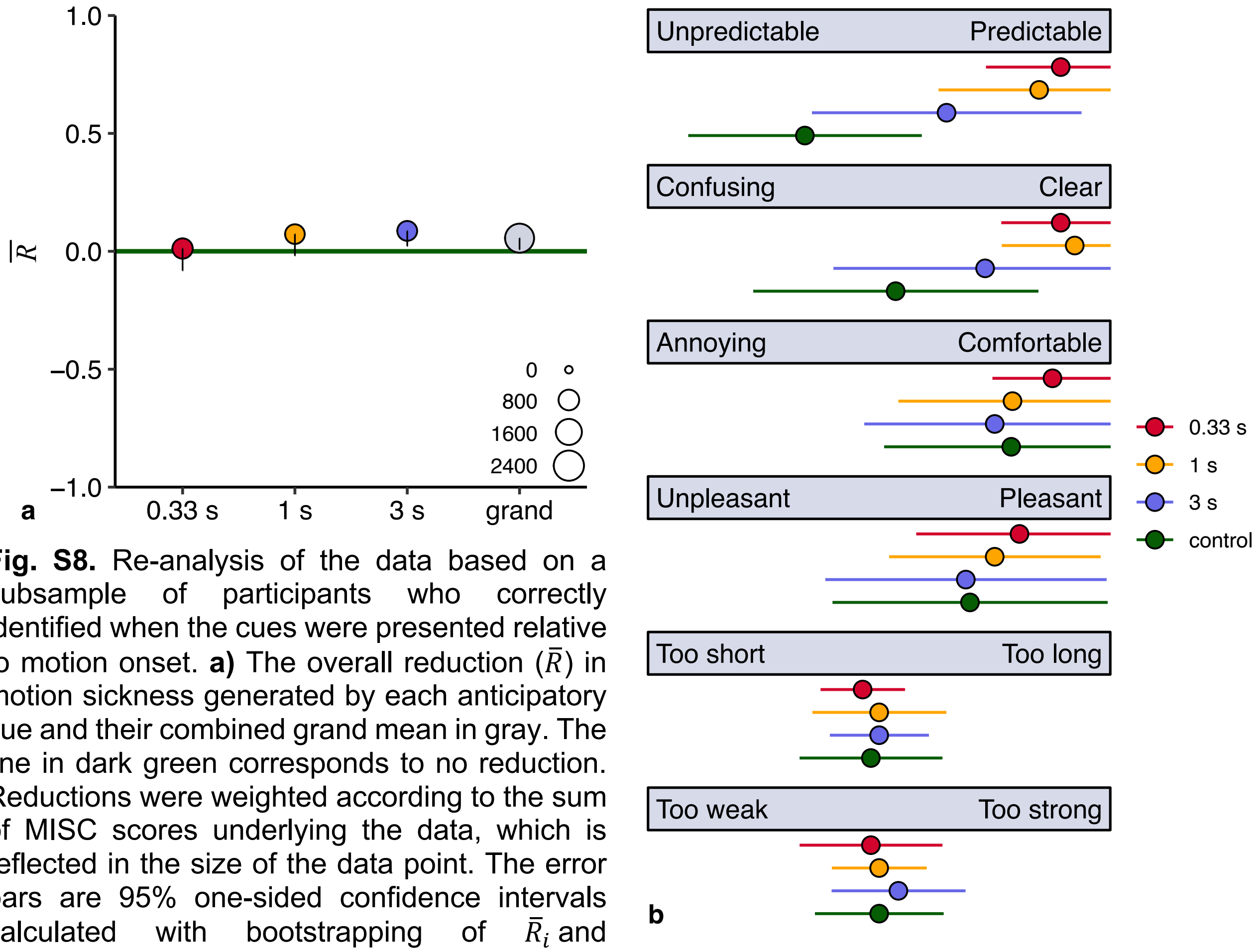

Fig. S8. Re-analysis of the data based on a subsample of participants who correctly identified when the cues were presented relative to motion onset. **a)** The overall reduction ($\overline{R}$) in motion sickness generated by each anticipatory cue and their combined grand mean in gray. The line in dark green corresponds to no reduction. Reductions were weighted according to the sum of MISC scores underlying the data, which is reflected in the size of the data point. The error bars are 95% one-sided confidence intervals calculated with bootstrapping of $\overline{R}_i$ and corresponding w_i values. 0.33 s: *n* = 14, 1 s: *n* = 13, 3 s: *n* = 12. **b**) Ratings along several user dimensions. Error bars indicate standard deviations

Comparison of MISC scores between the control sessions

Kuiper et al. (2020a) performed a comparable study on the effectiveness of anticipatory audio cues. Based on the overlapping standard deviations (shaded areas), we can conclude that the motion stimulus used in the current study is comparable in provocativeness compared to the study of Kuiper et al. (2020a).

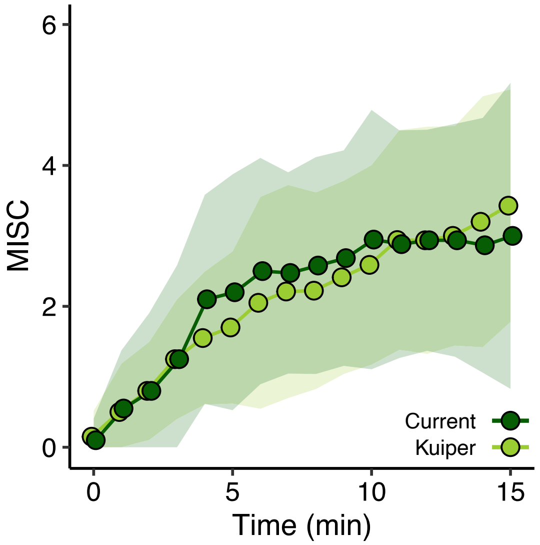

Fig. S9. Development of raw MISC scores in the control session of our current study in dark green and in the study of Kuiper et al. (2020a) in light green. The shaded areas represent standard deviations
